# Probabilistic divergence time estimation without branch lengths: dating the origins of dinosaurs, avian flight and crown birds

**DOI:** 10.1098/rsbl.2016.0609

**Published:** 2016-11

**Authors:** G. T. Lloyd, D. W. Bapst, M. Friedman, K. E. Davis

**Affiliations:** 1 Department of Biological Sciences, Macquarie University, New South Wales 2109, Australia; 2 Department of Geology, University of California Davis, One Shields Avenue, Davis, CA 94568, USA; 3 Department of Geology and Geological Engineering, South Dakota School of Mines and Technology, Rapid City, SD 57701, USA; 4 Department of Earth Sciences, University of Oxford, South Parks Road, Oxford OX1 3AN, UK; 5 Department of Biology, University of York, Wentworth Way, York YO10 5DD, UK

**Keywords:** birds, dinosaurs, divergence time, phylogeny

## Abstract

Branch lengths—measured in character changes—are an essential requirement of clock-based divergence estimation, regardless of whether the fossil calibrations used represent nodes or tips. However, a separate set of divergence time approaches are typically used to date palaeontological trees, which may lack such branch lengths. Among these methods, sophisticated probabilistic approaches have recently emerged, in contrast with simpler algorithms relying on minimum node ages. Here, using a novel phylogenetic hypothesis for Mesozoic dinosaurs, we apply two such approaches to estimate divergence times for: (i) Dinosauria, (ii) Avialae (the earliest birds) and (iii) Neornithes (crown birds). We find: (i) the plausibility of a Permian origin for dinosaurs to be dependent on whether *Nyasasaurus* is the oldest dinosaur, (ii) a Middle to Late Jurassic origin of avian flight regardless of whether *Archaeopteryx* or *Aurornis* is considered the first bird and (iii) a Late Cretaceous origin for Neornithes that is broadly congruent with other node- and tip-dating estimates. Demonstrating the feasibility of probabilistic time-scaling further opens up divergence estimation to the rich histories of extinct biodiversity in the fossil record, even in the absence of detailed character data.

## 1. Background

Divergence times are often estimated by combining fossil information with a phylogenetic hypothesis. In a classical clock-based ‘node-dating’ framework, a tree of extant taxa with branch lengths representing character changes is dated with reference to a set of fossil calibrations that constrain the minimum age for particular nodes. However, multiple important divergences within the tree of life are not bracketed by extant lineages (e.g. origin of birds) and thus cannot be estimated using molecular data. More recently, ‘tip-dating’ approaches have allowed extinct taxa to be included as terminals, with phylogenetic inference and divergence time estimation occurring simultaneously without reference to node calibrations. This opens up the possibility for using character change from molecular or morphological sources (or both) when estimating divergences between extinct and extant lineages, or even among entirely extinct lineages. Independently of these advancements, palaeontologists have been using stratigraphic ages to directly date divergences on existing phylogenies. Often constructed from both morphological cladograms and taxonomy, these lack measures of character change [1], with their strength instead relying on the stratigraphic distribution of fossils [2]. We term these ‘*a posteriori*’ time-scaling (APT) approaches.

Most of these APT approaches work independently of inferred amounts of character change (but see [3]), relying solely on occurrence data. Broadly speaking, three types are typically applied: (i) minimum-age dating [4], (ii) extending branch durations by adding a constant [5] and (iii) branch duration sharing [3]. The latter two utilize a minimum age (based on first appearances) dated tree as a preliminary step. These approaches suffer from arbitrary choices of required variables and make strong assumptions of the quality of the fossil record without reference to said fossil record. While Bayesian tip-dating methods have recently become accessible to completely extinct clades ([6]; other papers in Special Feature), their availability has also coincided with improvements in APT approaches [7].

Here, we assemble a novel phylogenetic hypothesis for Mesozoic dinosaurs and time-scale it using two different *probabilistic* APT methods—cal3 [7] and a new method developed from the node-dating approach of Hedman [8]. We use these results to ask three questions involving major evolutionary transitions: (i) When did Dinosauria originate?, (ii) When did birds originate? and (iii) How old is the avian crown? The first two involve dating the divergence of an extinct lineage, but the latter considers a split among extant taxa, thus permitting comparisons between our estimates and those from published clock-based analyses.

## 2. Material and methods

A novel ‘metatree’ approach (electronic supplementary material, figures S1, S2)—which operates in a similar way to formal supertree approaches, but generates source trees directly from character-taxon matrices rather than published figures (see the electronic supplementary material)—generated 1000 phylogenetic hypotheses containing 962 separate operational taxonomic units (OTUs). Of these, 100 were sampled at random to account for phylogenetic uncertainty (a larger number being computationally prohibitive). Ages came from fossil occurrence data in the Paleobiology Database and primary literature. Divergence times were then estimated for three nodes on the tree: (i) the origin of dinosaurs (inclusive and exclusive of the potential oldest dinosaur, *Nyasasaurus* [9]), (ii) the origin of Avialae (inclusive or exclusive of the purported earliest bird, *Aurornis* [10]) and (iii) the origin of crown birds (Neornithes).

We first applied the cal3 method of Bapst [7], which requires *a priori* estimates of diversification and sampling rates to draw likely divergence dates under a birth–death-sampling model and operates in a similar manner to the fossilized birth–death (FBD) process [11]. Sampling and extinction estimates were obtained by stochastically sampling sets of congruent taxon ranges from the occurrence data, via the function ‘seqTimeList’ in the R package palaeotree [12]. We calculated maximum-likelihood estimates of sampling and extinction rates using the resulting range frequency distributions [13], and used our extinction rate estimates as a proxy for speciation rate. To account for the uncertainty in these rate estimates, each cal3 tree was time-scaled with a different set of estimated rates. As the cal3 approach is stochastic, we applied it 1000 times and aggregated the results into distributions.

Next, we applied a novel algorithm based on the node-dating approach of Hedman [8]. Not all nodes can be appropriately dated using this algorithm (electronic supplementary material, figure S4), and thus, we utilized a novel approach to obtain missing dates via a randomization process (electronic supplementary material). Again, 1000 dates were estimated for each node on each tree. Thus for the three nodes distributions were produced from 100 000 point estimates for both the cal3 and Hedman approaches.

Tree searches were performed in TNT [14] and all other analyses in R [15].

## 3. Results

**Figure 1.**
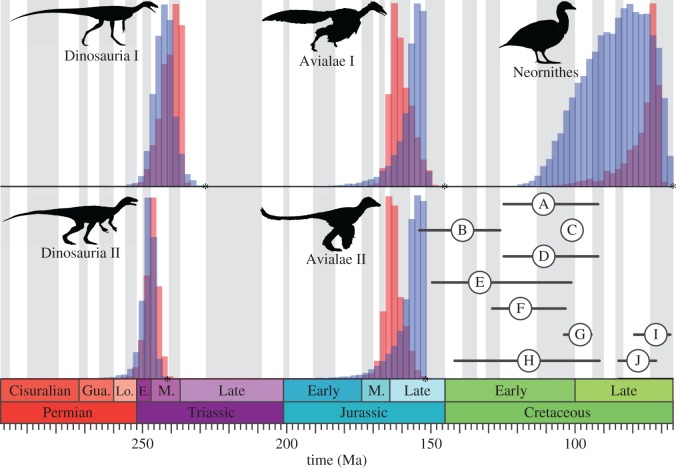
Comparison of probabilistic APT dates (red bars, cal3; blue bars, Hedman; see the text) for key nodes in dinosaur phylogeny: Dinosauria I (*Nyasasaurus* as sister to Dinosauria), Dinosauria II (*Nyasasaurus* nested within Dinosauria), Avialae I (*Archaeopteryx* as first bird), Avialae II (*Aurornis* as first bird) and Neornithes (crown birds). Asterisks mark minimum bound or ‘traditional’ palaeontological estimate. Molecular and morphological clock dates for Neornithes are shown in the lower right corner (A–J; electronic supplementary material, table S3): circles indicate mean and horizontal bars the 95% HPD. Silhouettes were taken from public domain images on phylopic.org (*Aurornis*, Gareth Monger; *Eoraptor*, Scott Hartman; *Vegavis*, Matt Martyniuk), or modified with kind permission from works by Sergio Pérez (*Archaeopteryx*) and Nobu Tamura (*Nyasasaurus*).

The main results are summarized in figure 1 and electronic supplementary material, table S2. Overall, the cal3 and Hedman approaches broadly agree (mean difference between median estimates = 5.4 Ma). However, the shapes of the distributions often vary (figure 1), with the Hedman approach giving less precise estimates (mean highest posterior density (HPD) width is 6.3 Ma greater than for cal3). The probability of a Permian origin for dinosaurs depends on whether *Nyasasaurus* is (2.6%, cal3; 9.6%, Hedman), or is not (0.2%, cal3; 1.0%, Hedman) considered a dinosaur. Conversely, the difference in the median age estimate is minimal regardless of whether *Archaeopteryx* or *Aurornis* is considered the earliest bird (2.8 Ma, cal3; 0.4 Ma, Hedman), despite a 7 Ma difference in their lower bounds. Finally, the 95% HPD width for crown birds is the largest for any node (23.4 Ma, cal3; 39.5 Ma, Hedman) due to both greater phylogenetic uncertainty and a poorer fossil record, creating overlap with multiple published clock-based estimates.

## 4. Discussion

Application of our metatree approach results in a well resolved strict consensus (electronic supplementary material, figure S3) which we attribute to the inclusion of a taxonomy ‘tree’ and our weighting scheme (see the electronic supplementary material), which create superior overlap and conflict resolution (compared with formal supertrees), respectively. Remaining topological uncertainty is distal to the main nodes under consideration, situated primarily among the long-necked sauropodomorphs and extinct bird clade Enantiornithes.

If *Nyasasaurus* is the oldest dinosaur, it significantly increases the age of the dinosaurian node. However, even if this taxon falls just outside the clade the upper 95% HPD does not exclude an Early Triassic age—an older value than most macroevolutionary studies apply [16]. Proper resolution of this positional uncertainty would require a larger archosaur phylogeny and illustrates the general difficulty of estimating root values. The absence of phylogenetic uncertainty at this node also coincides with the narrowest HPD width (greatest precision) and closest agreement between cal3 and Hedman estimates. This is to be expected as the major source of difference between cal3 and Hedman is the latter approach's greater sensitivity to the order of outgroups.

Although *Aurornis* may be a troodontid rather than a bird [17], either position does not seem to affect the estimated bird origin age here. Given the demonstrated volatility of dinosaur phylogeny [18], this is an encouraging result for the robustness of both approaches. The bird node also confirms that there is no consistent, and hence predictable, bias between both methods in terms of median age: here (unlike the root) cal3 returns the older median value.

Published age estimates for Neornithes vary considerably (figure 1). This uncertainty is in part due to a worse fossil record in crown- versus stem-birds, as well as the credibility of the neornithine affinities of some Late Cretaceous taxa [19] which make calibrating clock-based approaches difficult. Our methods also seem to capture this signal, as suggested by the largest HPD widths for both cal3 and Hedman. Both approaches also share the same level of accuracy as their clock-based comparisons: 95% HPDs overlap with all but one published estimate. Furthermore, both APT median age estimates fall within the range of mean clock-based ages. The neornithine node also illustrates the larger variability in distribution shape for the Hedman approach. This reflects the reliance on the order and age of outgroups that produce the distributions on which the Hedman approach is conditioned.

Overall both of our probabilistic APT approaches are broadly congruent with clock-based estimates and each other, suggestive of robustness. Thus, not only is it feasible to apply cal3 to vertebrate data (see also [20]), cal3 and Hedman should be preferred over APTs that ignore fossil record quality. Between these approaches and the FBD model [11], adequate divergence dating is available independently of whether lineages are still extant or have character information available. It is also conceivable that these distributions could inform priors on specific divergences in clock-based approaches, such as node- or tip-dating constraints. However, there is still room for improvement. Ideally, phylogenies dated with APT methods contain almost all taxa known from the fossil record, like the metatree constructed here, but this may be impossible for some groups. (For example, many invertebrate macrofossil species have never been included in a phylogenetic hypothesis [2].) Both APT approaches used here also make simplistic assumptions about sampling, which may vary considerably over time and space [21], and future approaches should relax such constraints as some implementations of the FBD model already allow. Regardless, the increasing ability to calculate robust divergence dates for phylogenies of fossil taxa opens more intersections on the tree of life to dating and subsequent macroevolutionary analyses.

## Supplementary Material

Figure S1

## Supplementary Material

Figure S2

## Supplementary Material

Figure S3

## Supplementary Material

Figure S4

## Supplementary Material

Supplementary Information

## Data accessibility

Source trees: graemetlloyd.com/matr.html; other data and code: http://dx.doi.org/10.5061/dryad.p660m [22].
