# Supplementary figures and images for: Probabilistic divergence time estimation without branch lengths: dating the origins of dinosaurs, avian flight and crown birds
Source: Biol Lett. 2016 Nov;12(11):20160609. doi: 10.1098/rsbl.2016.0609 (PMC5134040; doi:10.1098/rsbl.2016.0609)

Start:

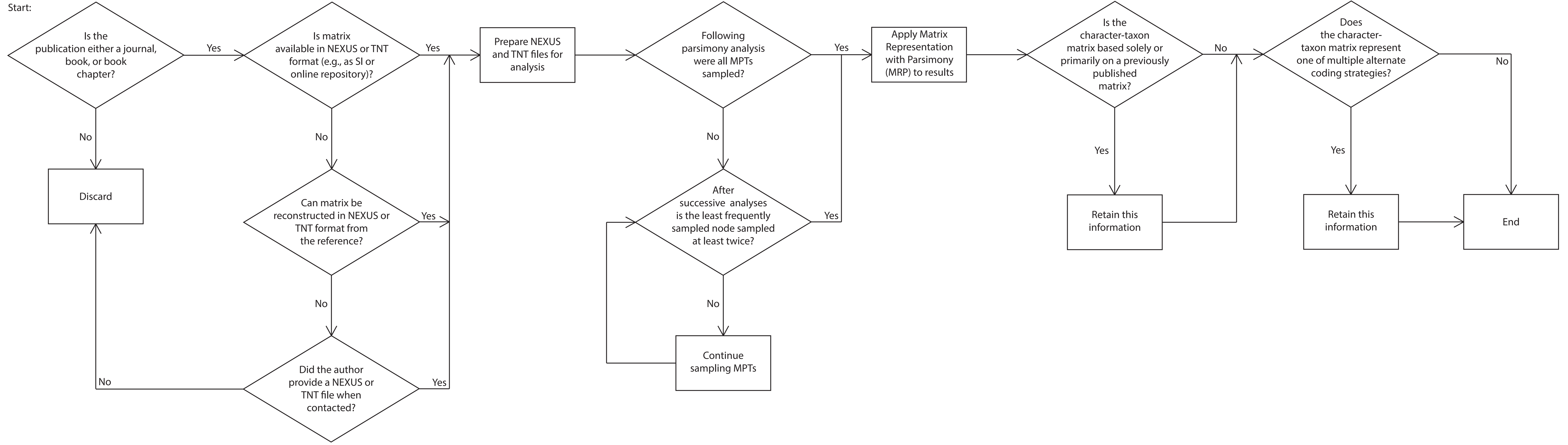

Supplement: Figure S1 [file rsbl20160609supp1.pdf]

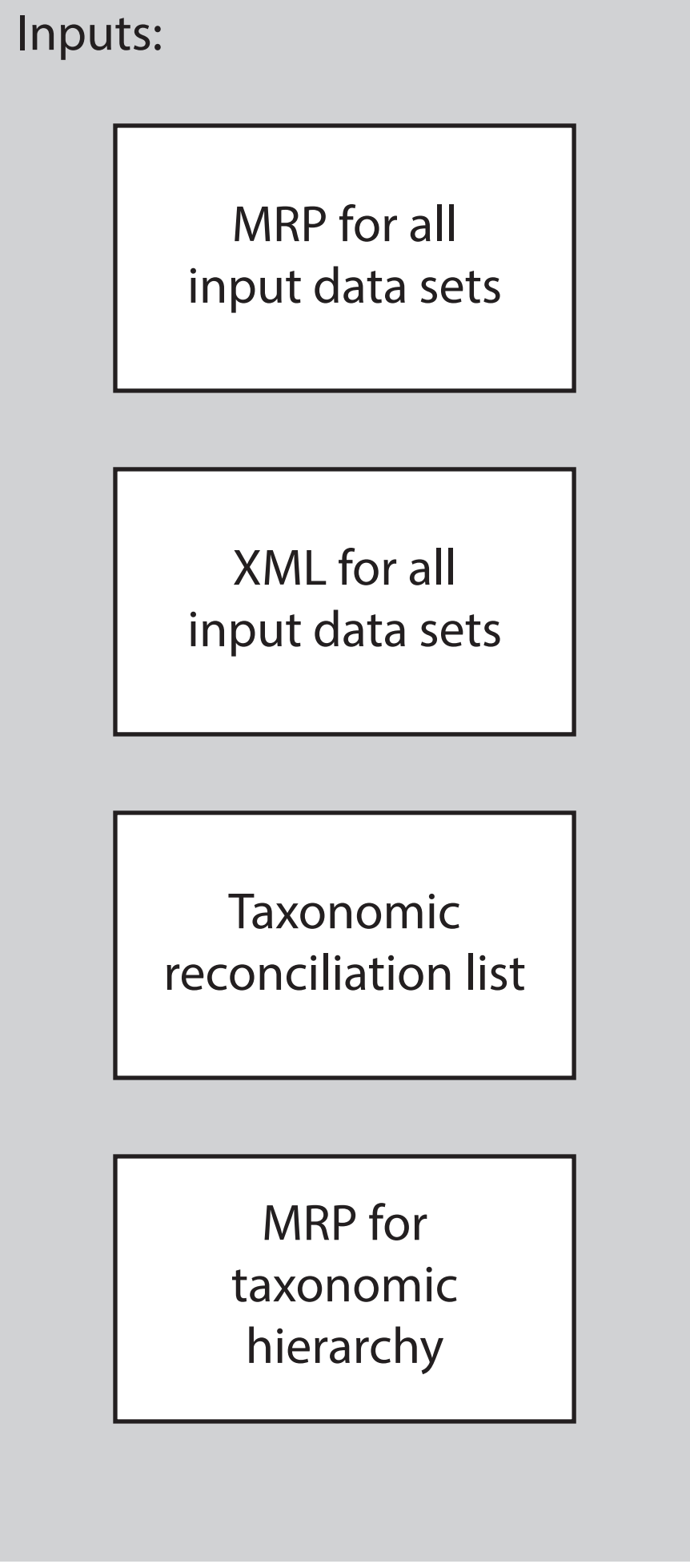

Start:

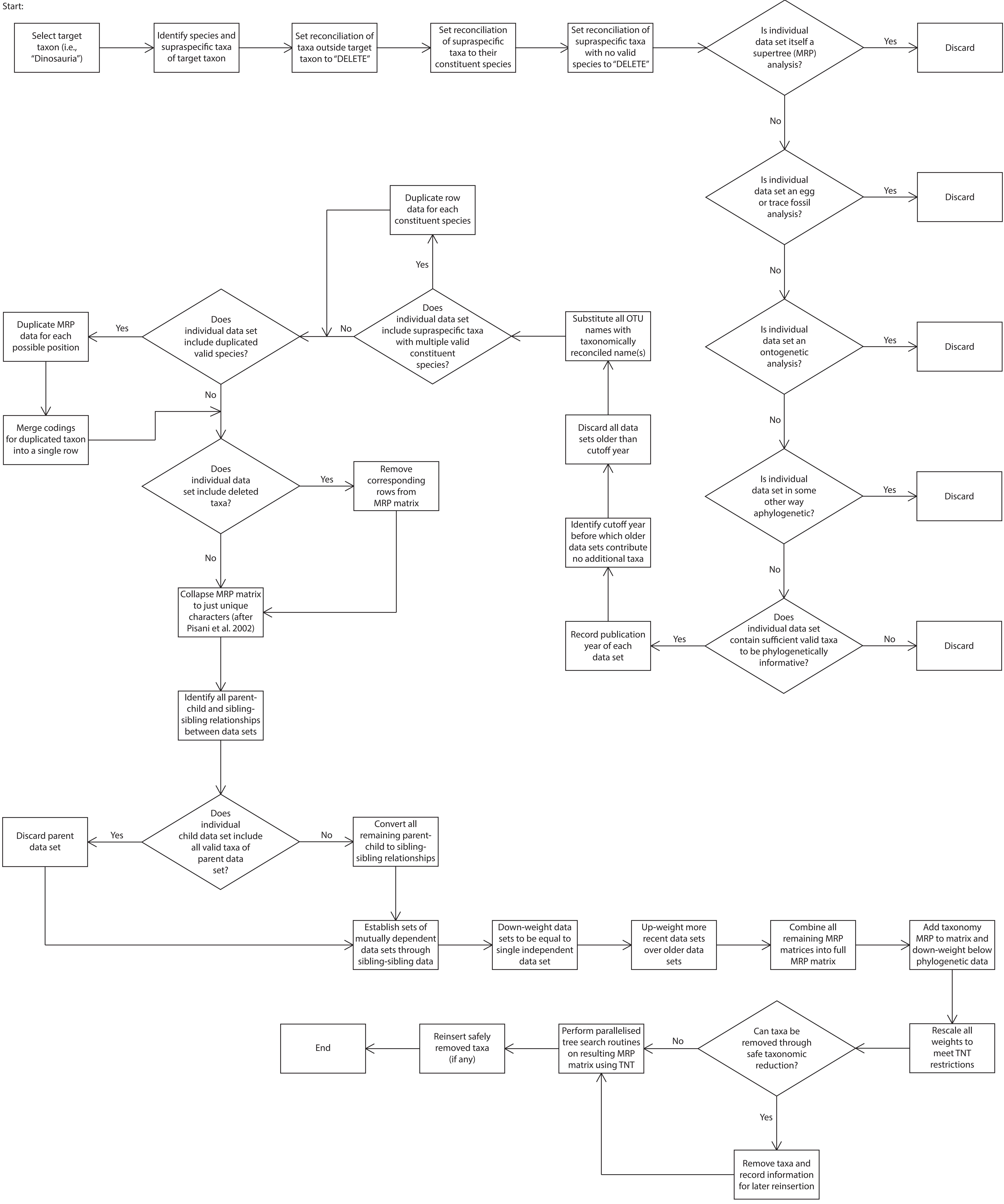

Supplement: Figure S2 [file rsbl20160609supp2.pdf]

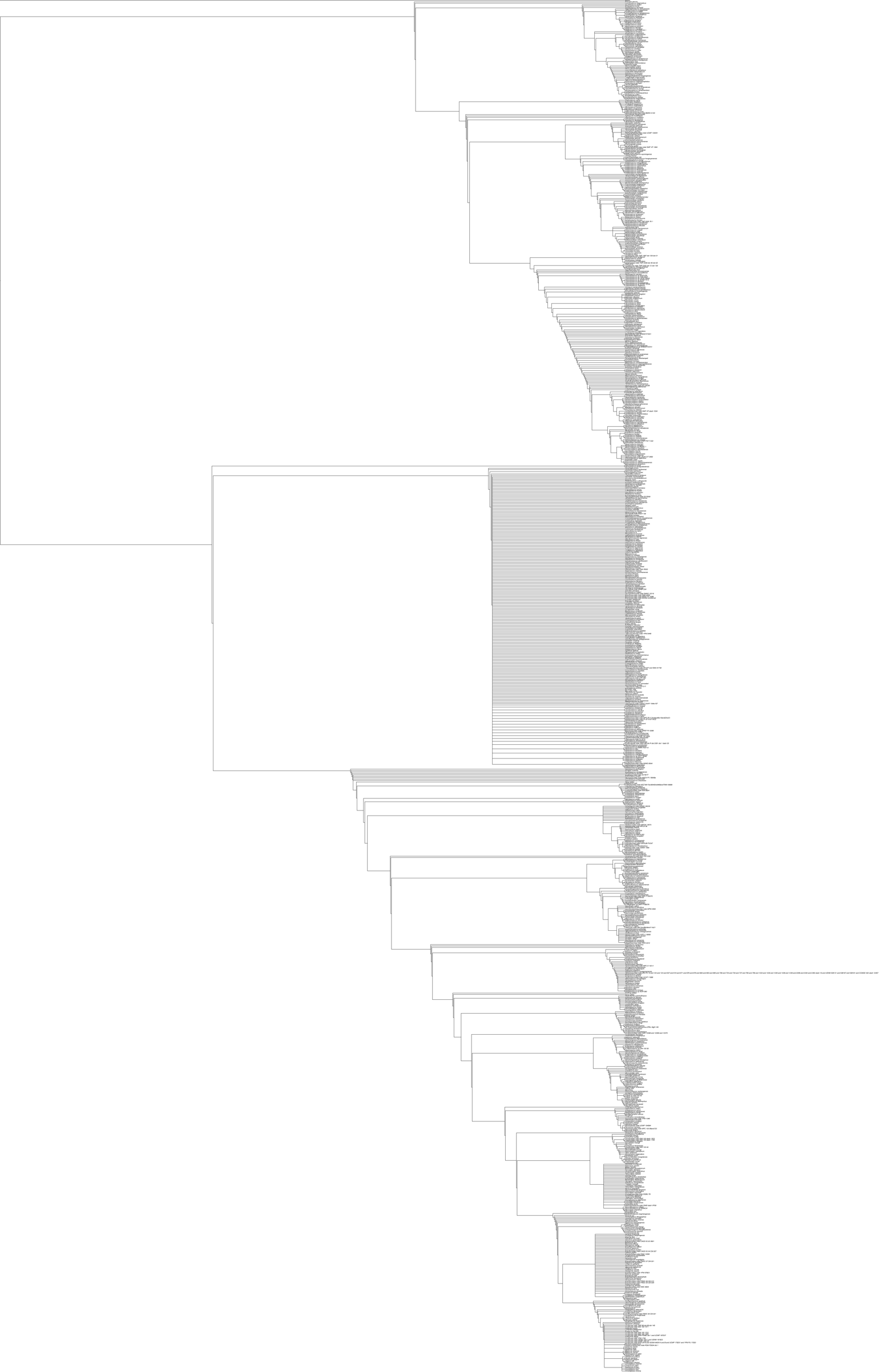

Supplement: Figure S3 [file rsbl20160609supp3.pdf]

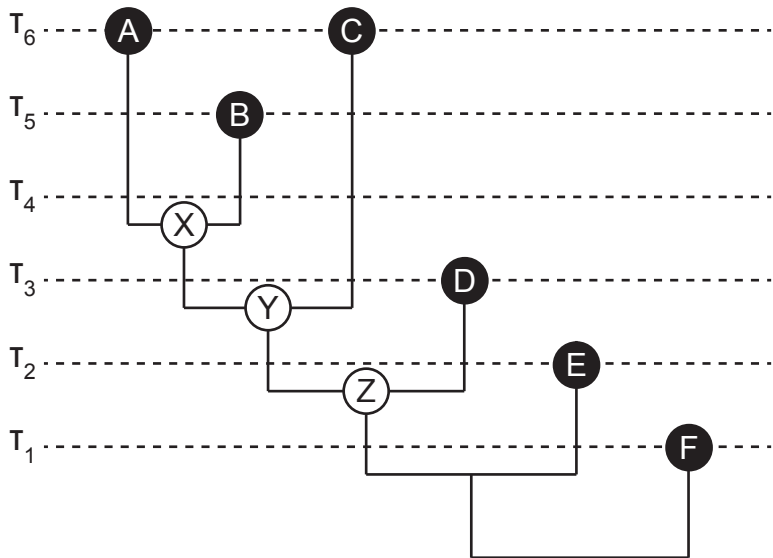

### Outgroup sequences

| Node | Standard |       |       |       |       | Conservative |       |       |       |
|------|----------|-------|-------|-------|-------|--------------|-------|-------|-------|
| (X)  | $T_5$    | $T_5$ | $T_3$ | $T_2$ | $T_1$ | $T_5$        | $T_3$ | $T_2$ | $T_1$ |
| (Y)  | $T_5$    | $T_3$ | $T_2$ | $T_1$ |       | $T_5$        | $T_3$ | $T_2$ | $T_1$ |
| (Z)  | $T_3$    | $T_2$ | $T_1$ |       |       | $T_3$        | $T_2$ | $T_1$ |       |

Supplement: Figure S4 [file rsbl20160609supp4.pdf]
