# Supplementary material for: Probabilistic divergence time estimation without branch lengths: dating the origins of dinosaurs, avian flight and crown birds
Source: Biol Lett. 2016 Nov;12(11):20160609. doi: 10.1098/rsbl.2016.0609 (PMC5134040; doi:10.1098/rsbl.2016.0609)
Supplement: Supplementary Information [file rsbl20160609supp5.docx]

**Supplementary Information for:**

**Probabilistic divergence time estimation without branch lengths: dating the origins of dinosaurs, avian flight, and crown birds**

Graeme T. Lloyd, David W. Bapst, Matt Friedman, and Katie. E. Davis

Contents:

1. Tree construction
2. Description of the Hedman algorithm
3. Probabilistic divergence dates
4. Clock-based dates for comparison
5. Dryad files
6. Supplementary References
7. Supplementary Figure Captions

**1) Tree construction**

*Source trees* – Unlike supertree approaches, which – regardless of whether they are formal or informal – take published trees as their input (i.e., figures from publications), our metatree approach generates source trees from published morphological character-taxon matrices, retaining their original character descriptions (e.g., ordered versus unordered) and weights.

Beginning with the list of Lloyd et al. (2008) we added substantial novel published cladistic analyses that contained at least three dinosaurian taxa (the minimal amount of phylogenetic information), updating their list (current to the end of 2006) to late 2013. A more up-to-date list inclusive of these trees is available from the first author’s web site (graemetlloyd.com/matr.html). Here the protocol of Lloyd et al. (2008) was followed (Figure S1) in that source trees were only considered if the data matrix used to produce them were made available, either directly from the original publication (including Supplementary Information and external repositories such as the author(s)’ web site, Treebase, or Dryad) or indirectly (obtained via email from the original author(s)). The importance of this step is to: 1) allow reanalysis of the matrices to standardise the input type (for example, Lloyd et al. 2008 used the strict component consensus (SCC) tree), 2) make sure all taxa are included (as figured trees often omit outgroups and other non-focal clades), and 3) ensure non-independence of data sets could be adequately accounted for (again, following Lloyd et al. 2008). In addition, this approach enables future research opportunities such as: 1) reanalysis of data sets using alternative optimality criteria (likelihood, Bayesian), 2) a first step towards an alternative supermatrix approach, and 3) generation of a large database of character information for meta-analytical studies (e.g., Wright et al. 2016). Traditional literature searches (Web of Knowledge, Google Scholar, reference lists of other dinosaur phylogeny papers) were supplemented by pre-existing collections of cladistics analyses (particularly Ross Mounce’s github repository: github.com/rossmounce/cladistic-data), electronic mailing lists (particularly the archives of the Dinosaur Mailing List: dml.cmnh.org), and social media (Facebook groups such as “WIKIPALEO” and Twitter). Some additional papers were also helpfully identified by Mickey Mortimer (theropoddatabase.com).

A potential criticism of the broader composite tree construction approach is that very old data sets are included alongside more recent works. However, in some cases the most recent phylogenetic hypothesis including a particular taxon may be quite old. Here we applied a pragmatic approach to obtain a balance between these two aims (ignoring outdated phylogenetic hypotheses, but maximizing taxonomic coverage). Specifically, the list of source trees was reduced by removing older trees that did not contribute to the total number of reconciled operational taxonomic units (OTUs). It is important that this step occur *after* taxonomic reconciliation so that we avoid including an older tree that contains a unique taxon if that taxon has since been synonymized or otherwise considered invalid (e.g., a nomen dubium). This was done by iteratively taking datasets from the most recent publication year to some earlier year until the number of OTUs is maximised. In practice this meant a cutoff of 1995, meaning source trees from 1994 and earlier were excluded.

This approach and the larger workflow are summarized in Figure S2.

*Metadata and data set independence* – After data collection each cladistic analysis was represented by: 1) a #NEXUS file (containing the original character scorings for each taxon), 2) a Hennig86/TNT file (containing the same information), 3) a Newick tree file (of all or the first 100,000 most parsimonious trees, whichever is smallest, generated from reanalysis of the Hennig86/TNT file in TNT; Goloboff et al 2008), 4) a Newick tree file (of the SCC tree of 3), 5) a Newick tree file (of just the first tree contained in 3), 6) an MRP representation of all most parsimonious trees (even those not included in 3, see below), and 7) an XML file (containing the metadata for the analysis). (See graemetlloyd.com/matr.html for examples.) This latter file contains bibliographic information on the data source, a list of taxonomic names for each OTU, information on the type of characters used, information on the trees procured (here this means the optimality criterion – all maximum parsimony – and the source: always reanalysis of the original matrix), the filename associated with each data set (effectively the primary key for the full database), and, finally, information on non-independence of the data.

This last piece of information is key to establishing both which data sets to retain and how to weight non-independent, non-redundant data (see below). Independence is difficult to quantify, but necessary due to the very high levels of data set reuse, where older data sets are revised, typically by augmenting them with additional taxa, characters, or both, or updating the scorings of previous coding schemes. Here a simple parent-child and sibling-sibling approach is used. A data set is considered a “parent” if it forms the sole or main source matrix for a subsequent cladistic analysis, and any such subsequent analysis is thus its’ “child”. A parent can have multiple children (e.g., Wilson 2002 is particularly “fecund”, being direct parent to 38 other data sets), but a child can only have a single parent. Typically, children supersede their parent as they contain all of the parent’s plus additional taxa, and thus the parent is discarded. Children that share a parent are siblings, but often cannot supersede each other as they typically have taxon sets that overlap, but both contain at least one unique taxon. Here they are down-weighted so they are considered to have equal weight to a single independent analysis (following Lloyd et al. 2008; see below). In some cases, taxa are pruned from the parent to create the child and in such cases the parent should be retained and is instead treated as a sibling. (In effect the key criterion for retention is that the taxon set not be redundant with respect to any child data set.) Direct sibling-sibling relationships can also exist due to alternative settings for tree searches. For example, alternate coding schemes (e.g., as in Farke et al. 2011) or runs where certain multistate characters are treated as ordered or unordered with no clear preference given by the author(s). These results are treated the same as the sibling-sibling relationships described above except that there is no parent data set with which to check for redundancy.

*Taxonomic reconciliation* – A key step before source trees can be subjected to metatree analysis is taxonomic reconciliation, i.e., the adherence to a single taxonomy of valid OTUs that allows matching of tips across data sets. This requires a single taxonomy to be applied across all source trees. Lloyd et al. (2008) made use of a (then) recent authoritative work (The Dinosauria, Second Edition; Weishampel et al. 2004) on dinosaur taxonomy, however, significant numbers of dinosaur taxa have been named since (e.g., Lloyd and Friedman 2013; their Figure 2) requiring a different, updated source. Here we use the “metataxonomy” provided by the Paleobiology Database (paleobiodb.org) and accessed through the Fossilworks portal (fossilworks.org). Although this is likely to suffer from inflated species numbers it does offer a dynamic taxonomy that is continuously updateable and will in future applications of our metatree approach be directly accessed through the Paleobiology Database API (Peters and McClennen in press).

Here, again following Lloyd et al. (2008), the species-level is preferred as this is the lowest level of the taxonomic hierarchy (thus avoiding the error common in many supertrees of nested taxa being included as separate OTUs), ensures monophyly of OTUs, maximizes information retention, and allows for later collapsing to supraspecific OTU-level if desired (a process that cannot be inverted for higher taxa). As with previous iterations of the dinosaur metatree (Pisani et al. 2002; Lloyd et al. 2008) some OTUs are retained that do not have formal species names. This decision was made on multiple grounds. Firstly, there is a clear precedent for such OTUs to later be given formal names. Secondly, such OTUs can be important in setting temporospatial limits (e.g., oldest member, southernmost member) for the larger clades to which they belong. This is particularly important for downstream analyses where trees are time-scaled (this study) or used for biogeographic analyses. Even if they cannot be retained for some downstream analyses, e.g., because they contain insufficient information for a desired trait, it is likely optimal to include them in an initial phylogenetic inference or time-scaling step and prune a posteriori if desired (see Sakamoto and Ruta 2012). Finally, because specimens have a formal naming system accidental duplication of OTUs can more effectively be avoided. This latter feature is established through a more sophisticated naming system for unnamed taxa than the previous dinosaur supertrees (Pisani et al. 2002; Lloyd et al. 2008) that instead adopted names such as “Quarry 9 coelurosaur” or “Bajo Barreal abelisaur”. Specifically, such OTUs are named in two parts, first their higher taxonomic assignment is established (e.g., *Baryonyx* sp., Abelisauridae indet.) then their specimen number(s) are appended (e.g., NHMUK R3741, MPCA 56). Effort was made to establish specimen numbers for every such OTU (particularly helpful here was the web site of Mickey Mortimer; theropoddatabase.com), but in some cases (e.g., “Brachiosauridae indet. MHNM Unreferred”, aka the “French *Bothriospondylus*”; Lapparent 1943) no registered specimen number could be found. Finally, to avoid downstream machine-readable issues all names are given with spaces (“ “) replaced by underscores (“_”) and special characters spelled out (e.g., “.” is replaced by “_dot_”).

In practice taxonomic reconciliation involves multiple steps. Firstly, valid species-level taxa are replaced by their full binomial. Secondly, valid specimen-level OTUs are replaced with a formal name using the process described above. Thirdly, invalid taxa such as nomina dubia, nomina nuda, non-dinosaurian taxa (for a discussion on *Nyasasaurus*; see below), and post-Mesozoic taxa are removed. Note that unlike the previous dinosaur supertrees (Pisani et al. 2002; Lloyd et al. 2008) Mesozoic birds were retained. Here we follow the protocol of Pisani et al. (2002; their figure 1) in pruning redundant nodes created by taxon deletions. Fourthly, junior synonyms were replaced by their senior counterparts. Note that in some cases this leads to duplicate OTU names. Where these occur and are monophyletic (the most common situation) they were collapsed to a single taxon. (For example, the Newick string “(Taxon_A,(Taxon_B,Taxon_B));” would become “(Taxon_A,Taxon_B);”.) Where they were polyphyletic (much rarer) all possible positions were considered and additional trees created to account for them. Fifthly, and finally, supraspecific OTUs were replaced by a polytomy with all valid species assigned to them that *also* occur as specific OTUs in at least one source tree. This latter choice ensures taxa are not included that have not been placed in a phylogenetic analysis and thus may obscure phylogenetic inference. This is particularly important when employing a metataxonomy as older, largely forgotten, taxa could otherwise be introduced.

Note that here we chose to include the problematic taxon *Nyasasaurus parringtoni* (see main text for a discussion) in the analysis as it potentially belongs within Dinosauria. However, a more appropriate approach to determining its phylogenetic position would require a comprehensive archosaurian metatree analysis that is deferred for a future publication.

*Input tree selection* – Whereas Lloyd et al. (2008) used the SCC tree obtained from parsimony reanalysis of the original data set as a single input tree here a major methodological leap is made in that all (i.e., not just the first 100,000) most parsimonious trees (MPTs) are used as input instead. There are several reasons to prefer this approach: 1) SCC trees are by definition devoid of critical information and frequently imply resolutions that are not optimal (Bell and Lloyd 2015; their Figure 4), 2) the additional information available in the MPTs can help either resolve clades (where data may otherwise be missing) or more correctly generate conflict and uncertainty (where different resolutions are found), and 3) a novel approach for encoding large amounts of trees (see below) makes this approach feasible where previously it would have produced unacceptably large amounts of input data. Note that although here all MPTs are chosen the same approach could be applied to a posterior sample of trees from a Bayesian analysis, either of the morphological data alone or combined morphological and temporal data (e.g., Lee et al. 2014a,b).

*Taxonomy as a source tree* – Other formal metatrees have employed taxonomies (represented as often poorly resolved trees where internal nodes represent nested sets of supraspecific taxa) as constraints to aid resolution where a complete lack of overlap exists. However, where phylogeny conflicts with taxonomy the phylogenetic data should perhaps more appropriately be followed as it is evidence-, rather than opinion-based. Thus here to aid resolution a taxonomic source tree, containing all the taxa (valid species and specimen-level OTUs, see above), was produced and included as an additional source tree. However, it was not used as a constraint and was down-weighted (see weights section below) by at least an order of magnitude below any phylogenetic source tree. Thus its information is only used when either: 1) there is no phylogenetic information available to resolve a specific node, or 2) conflicting data (with one “side” equivalent to taxonomy) requires a tie-break. Here the Paleobiology Database (fossilworks.org and paleobiodb.org) metataxonomy was used to establish the taxonomy source tree. This was edited manually to include the unnamed taxa (i.e., specimen-level OTUs). In the MRP matrix these are the last 290 characters.

*Matrix Representation with Parsimony (MRP) of source trees* – Lloyd et al. (2008) used MRP following the approach of Baum (1992) and Ragan (1992) to represent the SCC for each source tree, with each “character” representing an internal node (bipartition) of the tree, excluding the root (which would be a parsimony uninformative constant character). Here a new approach was used when performing tree searches on the original matrices that instead records internal nodes from across every single most parsimonious solution (an approach generalizable to other sets of trees such as the posterior sample of a Bayesian analysis). The previous impediment to this approach is the large number of MRP characters that would be created. For example, a single data set of 100,000 fully dichotomous MPTs of 100 taxa (a realistic value for multiple actual data sets) would generate 9.8 million MRP characters. However, the vast majority of these characters are mutually redundant. For example, any node found in the SCC will also be found in every MPT and hence will be duplicated 99,999 times in our example. However, this is also true for non-SCC nodes, which will usually be duplicated many, if not every, time. Thus by simply collapsing the number of MRP characters to those that are unique can dramatically reduce the total number to manageable sizes. The only remaining obstacle to this approach occurs when memory limits mean the total number of MPTs could not be fully sampled. For example, if tree searching is limited to 100,000 MPTs and that many trees are returned it suggests more exist and without further searches optimal clades could be missing from the MRP. This problem can be overcome by making an analogy to an ecological approach to sampling species richness. It can be argued that, using a species frequency distribution, once the *rarest species is sampled more than once* that all species have been sampled (Alroy 2010). The same approach can be used here such that if successive tree search results are collapsed to their unique MRP characters and appended to a master MRP matrix, where the frequency of those characters are recorded (as weights in a cladistic matrix), that once the lowest frequency is greater than one all possible nodes have been sampled. In practice this approach works very well, with the rarest MRP character usually occurring far in excess of one time, thus leading to great confidence that no additional unsampled node (bipartition) exists. (Unlike the ecological example this is a potential issue as we can never be completely sure we aren’t sampling the same tree twice if successive tree searches are required.) One exception to this was found in the real data, specifically the matrix of Kurochkin (1996), where 108,459 unique MRP characters were sampled before the search was abandoned. However, it should be noted that this is not a true cladistic matrix as the author did not subject it to formal analysis to generate the phylogenetic hypotheses shown in the paper (and it was thus excluded here; see Figure S2). Furthermore, applying this approach to a much large sample (1796 data sets; graemetlloyd.com/matr.html) found no further problematic data sets, suggesting this is a generalisable approach. In summary, MRP of MPTs allows recording of *every* equally optimal bipartition, even when all MPTs cannot be directly sampled due to memory limits. Thus the resulting MRP matrix could be subjected to parsimony analysis to return the full set of MPTs (memory permitting) that were used to generate it.

*Weights* – A common criticism of previous formal metatree approaches was that data sets are treated equally, even when there might be clear grounds for expecting the signal from some input trees to be superior than others. For example, Pisani et al. (2002) weighted all published trees in their analysis equally, even though they were a mixture of numerical analyses, hand-drawn, and second-hand phylogenetic hypotheses. Similarly, other recent metatrees (e.g., Brocklehurst et al. 2015) used figured trees from the original publication where these can represent different forms of consensus tree, a preferred tree, or a posteriori exclude (or simply not show) some taxa. Lloyd et al. (2008) introduced a simple form of weighting – to account for non-independence of source trees (see above). They also applied an approach that standardized the input tree type (a SCC) and excluded hand-drawn and second-hand trees (see above). However, two additional forms of weighting are applied here (Figure S2). The first is already described above, where a taxonomy is used as an additional source tree (but not a constraint) it was down-weighted (to 1) so it maximally has an order of magnitude less influence on the final topology than any phylogenetic source tree (minimum weight of 10). The second is a novel, but simple approach to weighing different data sets. Specifically, it weights data sets by their recentness, with a doubling of weighting every two years beginning with the oldest included analysis. Thus a simple equation gives the weight (*W*) for a given publication year (*x*):

|  | $W=2^{0.5(x-t_{0})}10$ | (1) |
| --- | --- | --- |

(Where *t_0_* is the publication year of the oldest included analysis and 10 is the smallest weight for a phylogenetic source tree.) The choice to double every two years was made so that the difference between successive years was not large (as in reality two papers could have been submitted at the same time but appear in print a year apart purely due to differences in journal publication times). However, by using a power term recent papers will far outweigh those from several years earlier. For example, in the equation given above a tree from 1995 would have 1/1024^th^ the weight of a tree from 2015. More generally, the assumption that our knowledge increases through study time, and more recent analyses should be considered superior to older analyses (when our collective knowledge of the dinosaur record was poorer), is here considered a reasonably “safe” one as, if violated, then it would have far worse broader implications.

In practice, then, three different weighting schemes are being applied simultaneously: 1) independent vs. non-independent analyses, 2) phylogenies vs. taxonomies, and 3) recent analyses vs. older analyses. Where, in each case, the former receives higher weight than the latter. An additional complication comes in how these different schemes should interact. In practice the taxonomy source tree can be separated from this problem (they are weighted one, the smallest value used), and it is only the independence and recentness weightings that need to be merged. Here, for simplicity, a simple product is used, but a more sophisticated approach can be envisaged for future metatrees. A final correction factor is required as the software used for tree searches (TNT; Goloboff et al. 2008) limits the maximum character weight to 1,000. However, this can effectively be extended to 31,000 by using the maximum number of character states (32) and forcing all characters to be treated as ordered. Additionally, TNT limits weights to two-decimal places and thus the final weighting scheme was forced into a 1.00 to 31,000.00 range.

*Safe Taxonomic Reduction (STR)* – Many previous metatrees (e.g. Pisani et al. 2002; Lloyd et al. 2008) suffer from poor resolution of their consensus trees, particularly the SCC, which is commonly a complete polytomy. This is due to some taxa being very poorly constrained in terms of their position. For example, they occur in only a single, small, source tree that is poorly resolved, thus they are coded for very few MRP characters and minimal information is provided for their phylogenetic position, allowing them to sit equally parsimoniously in widely varying parts of the tree. This is partly ameliorated here by using a taxonomy source tree that serves to reduce this uncertainty to varying degrees. (Using taxonomy as a constraint was another prior solution to this problem, but was not used here because of the arguments made above.) However, an additional step is to apply STR (Wilkinson 1995), which allows removal of the poorer constrained taxa prior to tree searches, provided their final position can be confidently inferred from their character codings. Previously such an attempt would be impossible due to taxon limits in the only available software (TAXEQ3; nhm.ac.uk/research-curation/research/projects/software). However, recently an R implementation without a taxon limit was provided by the R package Claddis (Lloyd 2016). Application of STR using Claddis allowed removal of 50 of 962 taxa (5%; listed in Table S1) prior to tree searches. This has the advantage of reducing the size of treespace that needs to be searched by *N* trees according to the equation:

|  | $N=\frac{1919!}{2^{959}1919!}-\frac{1809!}{2^{909}1809!}$ | (2) |
| --- | --- | --- |

(Where *N* is too large to actually calculate.)

**Table S1** - Taxa removed prior to tree searches under the principle of Safe Taxonomic Reduction (STR; Wilkinson 1995). The first column indicates the removed OTU, the second the retained OTU with which it is redundant, and the third column indicates the STR rule under which it was removed (see Wilkinson 1995). Note that some removed OTUs (e.g., “Dromaeosauridae_indet_DGBU_78”) are redundant with multiple retained OTUs and hence can most parsimoniously be reinserted in multiple positions in the recovered MPTs.

| Removed OTU | Retained OTU | Rule |
| --- | --- | --- |
| Abelisauridae_indet_AMNH_1955 | Majungasaurus_crenatissimus | Rule 2B |
| Acrotholus_audeti | Prenocephale_prenes | Rule 2B |
| Aeolosaurus_colhuehuapensis | Aeolosaurus_rionegrinus | Rule 2B |
| Alioramus_altai | Alioramus_remotus | Rule 2B |
| Apatornis_celer | Iaceornis_marshi | Rule 2B |
| Apatosaurus_excelsus | Apatosaurus_ajax | Rule 1B |
| Blasisaurus_canudoi | Arenysaurus_ardevoli | Rule 2B |
| Bonapartenykus_ultimus | Patagonykus_puertai | Rule 2B |
| Borogovia_gracilicrus | Troodon_formosus | Rule 2B |
| Brachiosauridae_indet_MPEF_PV_3099 | Brachiosaurus_altithorax | Rule 2B |
| Canardia_garonnensis | Aralosaurus_tuberiferus | Rule 2B |
| Daspletosaurus_sp_MOR_590 | Daspletosaurus_torosus | Rule 2B |
| Dicraeosaurus_sattleri | Dicraeosaurus_hansemanni | Rule 2B |
| Diplodocus_hallorum | Diplodocus_longus | Rule 2B |
| Dromaeosauridae_indet_DGBU_78 | Achillobator_giganticus | Rule 2B |
| Dromaeosauridae_indet_DGBU_78 | Adasaurus_mongoliensis | Rule 2B |
| Dromaeosauridae_indet_DGBU_78 | Atrociraptor_marshalli | Rule 2B |
| Dromaeosauridae_indet_DGBU_78 | Austroraptor_cabazai | Rule 2B |
| Dromaeosauridae_indet_DGBU_78 | Balaur_bondoc | Rule 2B |
| Dromaeosauridae_indet_DGBU_78 | Bambiraptor_feinbergorum | Rule 2B |
| Dromaeosauridae_indet_DGBU_78 | Buitreraptor_gonzalezorum | Rule 2B |
| Dromaeosauridae_indet_DGBU_78 | Deinonychus_antirrhopus | Rule 2B |
| Dromaeosauridae_indet_DGBU_78 | Dromaeosaurus_albertensis | Rule 2B |
| Dromaeosauridae_indet_DGBU_78 | Graciliraptor_lujiatunensis | Rule 2B |
| Dromaeosauridae_indet_DGBU_78 | Itemirus_medullaris | Rule 2B |
| Dromaeosauridae_indet_DGBU_78 | Linheraptor_exquisitus | Rule 2B |
| Dromaeosauridae_indet_DGBU_78 | Mahakala_omnogovae | Rule 2B |
| Dromaeosauridae_indet_DGBU_78 | Neuquenraptor_argentinus | Rule 2B |
| Dromaeosauridae_indet_DGBU_78 | Pyroraptor_olympius | Rule 2B |
| Dromaeosauridae_indet_DGBU_78 | Saurornitholestes_langstoni | Rule 2B |
| Dromaeosauridae_indet_DGBU_78 | Tianyuraptor_ostromi | Rule 2B |
| Dromaeosauridae_indet_DGBU_78 | Tsaagan_mangas | Rule 2B |
| Dromaeosauridae_indet_DGBU_78 | Utahraptor_ostrommaysi | Rule 2B |
| Dromaeosauridae_indet_DGBU_78 | Variraptor_mechinorum | Rule 2B |
| Dromaeosauridae_indet_DGBU_78 | Velociraptor_mongoliensis | Rule 2B |
| Dromaeosauridae_indet_DGBU_78 | Yurgovuchia_doellingi | Rule 2B |
| Edmontonia_rugosidens | Edmontonia_longiceps | Rule 1B |
| Eusauropoda_indet_HMN_MB_dot_R_dot_2091_dot_1_dash_30 | Mamenchisaurus_constructus | Rule 2B |
| Genyodectes_serus | Ceratosaurus_nasicornis | Rule 2B |
| Gravitholus_albertae | Colepiocephale_lambei | Rule 2B |
| Guildavis_tener | Apatornis_celer | Rule 1B |
| Guildavis_tener | Iaceornis_marshi | Rule 2B |
| Hesperornithiformes_indet_RSM_P2604_dot_1 | Brodavis_americanus | Rule 2B |
| Hexing_qingyi | Shenzhousaurus_orientalis | Rule 2B |
| Itemirus_medullaris | Velociraptor_mongoliensis | Rule 2B |
| Jiangshanosaurus_lixianensis | Alamosaurus_sanjuanensis | Rule 2B |
| Kritosaurus_horneri | Kritosaurus_navajovius | Rule 2B |
| Lambeosaurus_magnicristatus | Lambeosaurus_lambei | Rule 2B |
| Leyesaurus_marayensis | Adeopapposaurus_mognai | Rule 2B |
| Macrogryphosaurus_gondwanicus | Talenkauen_santacrucensis | Rule 2B |
| Miragaia_longicollum | Dacentrurus_armatus | Rule 2B |
| Mochlodon_suessi | Mochlodon_vorosi | Rule 2B |
| Nasutoceratops_titusi | Avaceratops_lammersi | Rule 2B |
| Ornithomimidae_indet_CMN_12068_and_12069_and_12070 | Ornithomimus_edmontonicus | Rule 2B |
| Orodrominae_indet_TMP_2008_dot_45_dot_02 | Oryctodromeus_cubicularis | Rule 2B |
| Pachycephalosauridae_indet_UCMP_130051 | Colepiocephale_lambei | Rule 2B |
| Pachycephalosauridae_indet_UCMP_130051 | Gravitholus_albertae | Rule 2B |
| Pachycephalosauridae_indet_UCMP_130051 | Hanssuesia_sternbergi | Rule 2B |
| Pegomastax_africanus | Manidens_condorensis | Rule 2B |
| Rebbachisauridae_indet_MIWG_6544 | Limaysaurus_tessonei | Rule 2B |
| Rebbachisauridae_indet_Pv_6718_67_MOZ | Rebbachisauridae_indet_MPS_RV_II_2to4and6to19and22to23 | Rule 2B |
| Rebbachisaurus_garasbae | Rayososaurus_agrioensis | Rule 2B |
| Sauroniops_pachytholus | Eocarcharia_dinops | Rule 2B |
| Stegosaurus_sp_LHNB_CN_1 | Stegosaurus_ungulatus | Rule 2B |
| Tataouinea_hannibalis | Demandasaurus_darwini | Rule 2B |
| Tetanurae_indet_MM_2and8to9and11to21 | Allosaurus_fragilis | Rule 2B |
| Titanosauria_indet_Fo_0014 | Opisthocoelicaudia_skarzynskii | Rule 2B |
| Titanosauria_indet_IGM_100_3005 | Sonidosaurus_saihangaobiensis | Rule 2B |
| Tyrannosauroidea_indet_UUVP_11689 | Albertosaurus_sarcophagus | Rule 2B |
| Tyrannosauroidea_indet_UUVP_11689 | Gorgosaurus_libratus | Rule 2B |
| Tyrannosauroidea_indet_UUVP_11689 | Tyrannosaurus_rex | Rule 2B |
| Unescoceratops_koppelhusae | Gryphoceratops_morrisoni | Rule 1B |
| Utahceratops_gettyi | Pentaceratops_sternbergii | Rule 2B |
| Yuanmousaurus_jiangyiensis | Mamenchisaurus_constructus | Rule 2B |
| Yunnanosaurus_robustus | Yunnanosaurus_huangi | Rule 2B |
| Zalmoxes_shqiperorum | Zalmoxes_robustus | Rule 2B |

*Tree searches* – After the above steps were performed an MRP matrix of 912 OTUs and 25,879 characters with 32 states and a range of character weights was generated in TNT (Goloboff et al. 2008) format (available from Dryad at <http://datadryad.org/resource/doi:10.5061/dryad.p660m>). A matrix of this size is not currently practical to analyse on a typical laptop or desktop machine and so tree searches were performed on a dedicated workstation, specifically a six core (Intel(R) Xeon(R) CPU E5-1650 v2 @ 3.50GHz) machine with 16Gb RAM. Tree searches were parallelised by running 1,000 separate replicates, each using the xmult 10 option in TNT (Goloboff et al. 2008). In each replicate a single tree was retained and after running for a few weeks 1,000 unique trees were found, all of equal length (12961437 steps). Assuming this is the optimal (shortest) length (and hence these are MPTs) all trees were taken forward for further analysis.

*STR reinsertion –* After tree searching was complete it was necessary to reinsert those taxa removed by STR (Table S1) as they are still potentially important contributors to divergence time estimation. This was done in two steps. First, those taxa that could only occupy one position on the tree (had a single sister taxon, either on their own, or collectively, forming a polytomy) were reinserted as such. Second, those taxa that could equally likely be the sister of two or more taxa were assigned a sister at random for each tree (i.e., the same sister was not used across all 1,000 trees) and reinserted next to it. After this step 1,000 equally optimal topologies including all 962 taxa (making it some 50% larger than the composite tree of Benson et al. 2014 - the largest current phylogenetic hypothesis) were produced and made ready for divergence time estimation. We note that most of the variation in these trees is within Sauropodomorpha, which collapse to an almost complete polytomy in an SCC (Figure S3).

*Temporal data for time-scaling -* For both cal3 and Hedman approaches (see below) it was necessary to supply tip ages to the algorithms. However, for most dinosaur taxa hard ranges were not possible to define as occurrences are frequently singletons assigned to a geologic stage (or worse, e.g., an epoch). Thus temporal uncertainty for tips is high. One common way to deal with this problem is to randomly assign a date to a taxon using the limits of its age as the bounds of a uniform distribution, then repeat the process several times to gauge the effects of this uncertainty. However, as both the cal3 and Hedman algorithms are considerably slower than the simpler APT dating algorithms offered in paleotree (Bapst 2012) and strap (Bell and Lloyd 2015) this approach was not considered viable here. Significantly, though, our objective here was to establish divergence time estimates only and not infer branch lengths. Thus an approach was applied whereby we simply used the hard lower bound for each taxon as our tip age. In practice this meant for each taxon assembling the oldest and youngest possible age for each occurrence then taking the oldest of the youngest possible ages as the point by which the taxon *must* have evolved. This potentially biases our estimates towards younger dates, but avoids the possible pitfall of particularly uncertainly dated taxa having a strong influence on the final outcome.

*Selecting trees for dating* - Although we wished to account for phylogenetic uncertainty there were two limiting factors. Firstly, sampling additional MPTs (which almost certainly exist) was computationally prohibitive in terms of memory, thus we limited our search to the 1,000 trees retained from out TNT (Goloboff et al. 2008) searches. Secondly, running both cal3 and Hedman approaches (see below) added an additional practical limit in terms of computation time. We thus reduced our 1,000 sampled trees to 100 trees (selected at random and available from Dryad at <http://datadryad.org/resource/doi:10.5061/dryad.p660m>) prior to dating. This does not guarantee that our sample adequately captures the true variation across equally optimal topologies, but is a considerably better choice than using a single tree (especially an SCC, see arguments in Bell and Lloyd 2015).

*Defining nodes for dating* - As we were examining the age of key nodes across different topologies it was necessary to define these nodes in a way that would be flexible enough to work where relationships differed. In practice the nodes used were: 1) for Dinosauria simply the root (with or without *Nyasasaurus* included as a tip), 2) for Avialae inclusive of *Aurornis* the most recent common ancestor (MRCA) of *Aurornis xui* and *Vegavis iaai*, 3) for Avialae exclusive of *Aurornis* the MRCA of *Archaeopteryx lithographica* and *Vegavis iaai*, and 4) for Neornithes the MRCA of *Palintropus retusus*, *Polarornis gregorii*, *Torotix clemensi*, and *Vegavis iaai*. In this last case all four taxa were both included in our metatree and taxonomically assigned to Neornithes in the literature, thus they exemplify the crown. However, it should be noted that although multiple Cretaceous fossil occurrences have been assigned to Neornithes there remains some controversy over all such interpretations (Smith and Ksepka 2015). This has lead, for example, Prum et al. (2015) in their divergence time estimations to consider both a latest Cretaceous and a Paleogene age for the first definitive neornithine. Here we simply retain the current published assignments of these taxa, but note that this is an issue that may warrant further future examination.

**2) Description of the Hedman algorithm**

Our algorithm is a whole-tree extension of that first presented in Hedman (2010) for dating a single node, thus is hereafter referred to as WTEHA (Whole Tree Extension of the Hedman Algorithm). Hedman’s approach is a simple Bayesian method for estimating the divergence time for a single node that uses a set of uniform priors representing successive outgroup taxa. These can differ in their lower bound, but share a common (and arbitrary) upper bound. In practice young estimates will be found if successive outgroups have lower bounds very close to that for the node in question, i.e., if outgroups are all the same (or very close) age as the oldest member of the ingroup. Whereas large gaps between outgroup taxa imply a “gappier” record and older estimate. Critically, Hedman’s (2010) approach (unlike other APT methods) provides a posterior distribution of ages that can be both visualised and interrogated for an average and confidence intervals. Here we exclusively use the “conservative” approach proposed by Hedman (2010) and ignore dates that are younger than the preceding outgroup taxon (see Figure S4 for a visual explanation of the difference). This is because the non-conservative approach is biased towards minimum estimates (see node X in Figure S4) in the same way as pre-existing minimal time-scaling algorithms are (Smith 1994; Ruta et al. 2006; Brusatte et al. 2008). However, the current R implementation (available at: graemetlloyd.com/pubdata/functions_7.r as Hedman.tree.dates) can perform both approaches allowing the user this option. Use of this conservative approach has the potential to make the successive outgroups to a given node non-unique (compare nodes X and Y in Figure S4), meaning that successive nodes can lead to the same divergence estimate. Left unmodified such instances imply the instantaneous diversification and division-by-zero problems caused by the minimal algorithms of Norell (1992) and Smith (1994). This is not purely a hypothetical problem, as it is found to be the case in all of the phylogenies examined here. Thus the (conservative) Hedman (2010) approach cannot be extended without modification.

We began our modification by taking the R function provided in the appendix to Hedman (2010) – that implements the Hedman algorithm (HA hereafter) – to work with the positive age values typically used by palaeontologists (and not the negatives implied by preceding time). WTEHA proceeds by first identifying the nodes that can correctly be dated using the HA, this is the most deeply nested node with a specific outgroup sequence. In the example of Figure S4 this would be nodes X and Z. Note that all “cherries” – nodes leading to two tips – will necessarily be dated using the HA. (This has a desirable property of meaning that at the next step - where nodes are dated using HA-dated bounding nodes - there is always a lower bound. In other words there are never nodes too young to not have a HA-dated node that is directly or indirectly a descendant.) For each of these nodes the age of its successive outgroups are calculated and appended by the outgroup sequence to the whole tree (see below) and the absolute maximum upper bound (*t_0_*, here we conservatively used the base of the Cambrian, 542 Ma) is also supplied. These values are then fed to the otherwise unmodified HA and the posterior distributions of ages that are returned are then stored for later use. Dating the nodes close to, and inclusive of, the root creates a separate problem where there are insufficient outgroup ages for the HA to function. Thus to use the WTEHA an additional sequence of outgroup ages from outside of the current tree must be provided.. Here we used the following sequence of ages (in order of increasing proximity to Dinosauria): 315 Ma, 304 Ma, 290 Ma, 265 Ma, 260 Ma, 260 Ma, 252 Ma, 250 Ma, 250 Ma, and 247 Ma. These are based primarily on the relationships in Nesbitt (2011) and were added as tips to the trees supplied to cal3 in order that the comparisons be fair. At this stage all HA-dateable nodes have been dated.

WTEHA then identifies the remaining undated nodes, but first an additional step is used to ask whether the root of the tree has been HA-dated. If it has then all remaining nodes must be constrained (above and below) by HA-dated nodes. I.e., all remaining undated nodes *must* be bounded by HA-dated nodes. If not then an extra step is performed whereby a dummy node is inserted just prior to the root and dated using the HA and *just* the outgroup sequence from outside the tree. After this step all undated nodes must be constrained above and below by HA-derived posterior distributions.

WTEHA then identifies dependent sets of nodes that are constrained by a common set of HA-dated nodes, e.g., a series of ancestor-descendant nodes between two HA-dated nodes. The specific constraining nodes are also identified at this point. In every case the upper bound must be a single node (a single common ancestor), but the lower bound can potentially be two or more nodes (if their posterior distributions overlap, as they typically do). Constraining dates are then drawn at random from the posterior distributions of the constraining nodes, with the maximum date from the lower bounding nodes being used as the lower constraint if there are two or more of them. If the constraining dates conflict (the maximum bound date is less than the lower bound date) then dates are redrawn until the maximum bound date is greater than the lower bound date. This step introduces a bias towards drawing dates from the lower end of the lower bounding distribution(s) and the upper end of the upper bounding distribution. Without intervention this can lead to distributions for non-HA-dated nodes that are skewed, with medians that conflict in age with the bounding HA-dated nodes (i.e., implying ancestors being younger than descendants and vice versa). Thus after half of the replicates are complete (the last possible stage an intervention can be made) an additional conditional is introduced that asks whether the medians of the distributions drawn at that point conflict in age order. If they do then random dates are drawn from the upper half of lower bounding distribution(s) and the lower half of upper bounding distributions until the conflict is resolved. This overcomes the bias and ensures that median ages of distributions will not conflict in age order, i.e., an ancestor will always be older than its descendant(s).

At this point upper and lower bounding ages have been drawn and now ages for the intervening non-HA-dated nodes can be drawn. These are drawn from a uniform (flat) distribution bounded by the upper and lower constraining dates, with the number of dates drawn equal to the number of intervening nodes. These randomly drawn dates are then sorted and assigned to the nodes in their correct sequence (i.e., the oldest date is assigned to the least nested node and the rest in sequence until the the youngest date is assigned to the most nested node). This process is repeated for as many replicates as the user dictates (here 1,000) until the intervening nodes have posterior distributions populated to the same size as the HA-dated nodes. These posterior distributions are stored alongside the HA-dated posteriors giving a complete set of posterior distributions for each node in the tree.

Finally, the function returns these distributions alongside a summary table that incorporates the median, 95% confidence intervals (upper and lower values) of each distribution alongside a simple binary value indicating whether that node was dated using the HA (indicated by a “1”) or by the WTEHA randomisation process (indicated by a “0”). In addition, a summary phylogenetic tree in “phylo” format is also returned with branch lengths scaled to time using the median dates for each node. Note that we present the median age of the distribution as our “best” estimate rather than the more commonly used mean of molecular workers (e.g., Meredith et al. 2011) as it is less likely to be skewed by the lengthy tails of some posterior distributions.

**3) Probabilistic divergence dates**

Median and 95% HPD probabilistic dates (cal3 and WTEHA) for the nodes shown in Figure 1 are presented in Table S2.

**Table S2** - Summary statistics for the probabilistic divergence dates for the nodes shown in Figure 1. The first column is the node name (exc. = excluding, inc. = including), the second column indicates the method used, the third column is the median estimate, and the fourth and fifth columns show the 95% highest posterior density (HPD) values.

| Node | Dating Method | Median | Lower 95% HPD | Upper 95% HPD |
| --- | --- | --- | --- | --- |
| Dinosauria I  (exc. *Nyasasaurus*) | cal3 | 240.3 Ma | 236.4 Ma | 248.3 Ma |
|  | Hedman | 243.0 Ma | 236.0 Ma | 249.8 Ma |
| Dinosauria II  (exc. *Nyasasaurus*) | cal3 | 247.5 Ma | 243.6 Ma | 252.0 Ma |
|  | Hedman | 248.7 Ma | 245.5 Ma | 255.1 Ma |
| Avialae I  (*Archaeopteryx*) | cal3 | 160.8 Ma | 150.8 Ma | 165.8 Ma |
|  | Hedman | 155.9 Ma | 152.1 Ma | 171.4 Ma |
| Avialae II  (*Aurornis*) | cal3 | 163.6 Ma | 155.8 Ma | 168.3 Ma |
|  | Hedman | 156.3 Ma | 152.4 Ma | 172.7 Ma |
| Neornithes | cal3 | 73.7 Ma | 70.7 Ma | 94.1 Ma |
|  | Hedman | 84.8 Ma | 69.1 Ma | 108.6 Ma |

**4) Clock-based dates for comparison**

Comparative molecular and morphological clock-based dates (A-J in Figure 1) were taken from several sources (Table S3). Specifically, multiple older molecular clock dates were provided by van Tuinen (2009; A-F) and were supplemented by those from two recent papers (Jarvis et al. 2014; Prum et al. 2015; H, I, and J) as well as a morphological clock estimate (Lee et al. 2014a; G). This final estimate is thus a tip-dating one, whereas all others are node-dating ages.

**Table S3** - Clock-based (molecular and morphological) estimates for the age of crown birds (Neornithes) taken from the literature and plotted in Figure 1. The first column indicates to which value in Figure 1 the estimate refers, the second column the actual estimate, the third column the maximum (95%) HPD, the fourth column the minimum (95%) HPD, and the final column the source of the dates.

| Figure 1 | Estimate | Max HPD | Min HPD | Source |
| --- | --- | --- | --- | --- |
| A | 111.0 | 125.0 | 96.0 | Node 1 from “Ref. 21” in Table 1 in van Tuinen (2009), after Paton et al. (2002). |
| B | 139.0 | 154.0 | 126.0 | Node 1 from “Ref. 22” in Table 1 in van Tuinen (2009), after Pereira et al. (2006). |
| C | 110.6 | 125.0 | 96.0 | Node 1 from “Ref. 23” in Table 1 in van Tuinen (2009), after Harrison et al. (2004) |
| D | 101.0 | - | - | Node 1 from “Ref. 24” in Table 1 in van Tuinen (2009), after Slack et al. (2006). |
| E | 133.2 | 149.0 | 115.0 | Node 1 from “Ref. 25” in Table 1 in van Tuinen (2009), after Brown et al. (2008). |
| F | 119.0 | 129.0 | 108.0 | Node 1 from “Ref. 29” in Table 1 in van Tuinen (2009), after Dyke and van Tuinen (2004). |
| G | 115.9 | 141.9 | 91.7 | “Crown Aves” uncorrelated clock age for “root: hard upper bound 200 Ma” in Table 1 from Lee et al. (2014a). |
| H | 98.4 | 103.0 | 94.5 | Supplementary Information date for “Neornithes” from Jarvis et al. (2014). |
| I | 72.7 | 79.5 | 67.0 | Supplementary Information date for “avian crown clade” without *Vegavis* from Prum et al. (2015). |
| J | 78.3 | 84.9 | 72.0 | Supplementary Information date for “avian crown clade” with *Vegavis* from Prum et al. (2015). |

**5) Dryad files**

*dinosaur_MRP.tnt* - The MRP matrix submitted to TNT (Goloboff et al. 2008) for performing tree searches; in Hennig86/TNT format.

*dinosaur_tnt_1000_mpts.tre.zip* - The 1,000 MPTs returned from the TNT tree searches; in Newick format (and zipped).

*dinosaur_str_1000_mpts.tre.zip* - The 1,000 MPTs returned from the TNT tree searches with STR taxa reinserted; in Newick format (and zipped).

*dinosaur_dating_100_mpts.tre* - The 100 trees randomly sampled and taken forward for dating; in Newick format.

*consensus.tre* – Strict consensus of the 1,000 MPTs of *dinosaur_str_1000_mpts.tre.zip* in Newick format.

*dinosaur_occurrences.xlsx* - The dinosaur occurrences used for dating; in MS Excel format.

*dinosaur_occurrences.txt* - The dinosaur occurrences used for dating; in plain text format.

*dinosaur_tipages.txt* - The tip ages (hard lower bounds) used for dating; in plain text format.

*dinosaur_timelist.txt* - The time list data used to generate the three rates used in the cal3 function; in plain text format.

*cal3_rates_code.R* - The R code used to generate the three rates used in the cal3 dating code; in R code format.

*cal3_dating_code.r* - The R code used to perform the cal3 dating; in R code format.

*Hedman_code.r* - The R code used to perform the Hedman dating; in R code format.

*cal3_dates.txt* - The cal3 dates output from R; in plain text format.

*Hedman_dates.txt* - The Hedman dates output from R; in plain text format.

All files are available from Dryad at <http://datadryad.org/resource/doi:10.5061/dryad.p660m>.

**6) Supplementary References**

Alroy, J., 2010. The shifting balance of diversity among major marine animal groups. *Science*, **329**, 1191-1194.

Bapst, D. W., 2012. paleotree: an R package for paleontological and phylogenetic analyses of evolution. *Methods in Ecology and Evolution*, **3**, 803-807.

Baum, B. R., 1992. Combining trees as a way of combining data sets for phylogenetic inference, and the desirability of combining gene trees. *Taxon*, **41**, 3-10.

Bell, M. A. and Lloyd, G. T., 2015. strap: an R package for plotting phylogenies against stratigraphy and assessing their stratigraphic congruence. *Palaeontology*, **58**, 379-389.

Benson, R. B. J., Campione, N. E., Carrano, M. T., Mannion, P. D., Sullivan, C., Upchurch, P. and Evans, D. C., 2014. Rates of dinosaur body mass evolution indicate 170 million years of sustained ecological innovation on the avian stem lineage. *PLoS Biology*, **12**, e1001853.

Brocklehurst, N., Ruta, M., Muller, J. and Frobisch, J., 2015. Elevated extinction rates as a trigger for diversification rate shifts: early amniotes as a case study. *Scientific Reports*, **5**, 17104.

Brown, J. W., Rest, J. S., Garcia-Moreno, J., Sorenson, M. D. and Mindell, D. P., 2008. Strong mitochondrial DNA support for a Cretaceous origin of modern avian lineages. *BMC Biology*, **6**, 6.

Brusatte, S. L., Benton, M. J., Ruta, M. and Lloyd, G. T., 2008. Superiority, competition, and opportunism in the evolutionary radiation of dinosaurs. *Science*, **321**, 1485-1488.

Dyke, G. J. and van Tuinen, M., 2004. The evolutionary radiation of modern birds (Neornithes): reconciling molecules, morphology and the fossil record. *Zoological Journal of the Linnean Society*, **141**, 153-177.

Farke, A. A., Ryan, M. J., Barrett, P. M., Tanke, D. H., Braman, D. R., Loewen, M. A. and Graham, M. R., 2011. A new centrosaurine from the Late Cretaceous of Alberta, Canada, and the evolution of parietal ornamentation in horned dinosaurs. *Acta Palaeontologica Polonica*, **56**, 691-702.

Goloboff, P. A., Farris, J. S. and Nixon, K. C., 2008. TNT, a free program for phylogenetic analysis. *Cladistics*, **24**, 774-786.

Harrison, G. L., McLenachan, P. A., Phillips, M. J., Slack, K. E., Cooper, A. and Penny, D., 2004. Four new avian mitochondrial genomes help get to basic evolutionary questions in the Late Cretaceous. *Molecular Phylogenetics and Evolution*, **21**, 974-983.

Hedman, M. M., 2010. Constraints on clade ages from fossil outgroups. *Paleobiology*, **36**, 16-31.

Jarvis, E. D., Mirarab, S., Aberer, A. J., Li, B., Houde, P., Li, C., Ho, S. Y. W., Faircloth, B. C., Nabholz, B., Howard, J. T., Suh, A., Weber, C. C., Fonseca, R. R., Li, J., Zhang, F., Li, H., Zhou, L., Narula, N., Liu, L., Ganapathy, G., Boussau, B., Bayzid, M. S., Zavidovych, V., Subramanian, S., Gabaldón, T., Capella-Gutiérrez, S., Huerta-Cepas, J., Rekepalli, B., Munch, K., Schierup, M., Lindow, B., Warren, W. C., Ray, D., Green, R. E., Bruford, M. W., Zhan, X., Dixon, A., Li, S., Li, N., Huang, Y., Derryberry, E. P., Bertelsen, M. F., Sheldon, F. H., Brumfield, R. T., Mello, C. V., Lovell, P. V., Wirthlin, M., Schneider, M. P. C., Prosdocimi, F., Samaniego, Jé. A., Velazquez, A. M. V., Alfaro-Núñez, A., Campos, P. F., Petersen, B., Sicheritz-Ponten, T., Pas, A., Bailey, T., Scofield, P., Bunce, M., Lambert, D. M., Zhou, Q., Perelman, P., Driskell, A. C., Shapiro, B., Xiong, Z., Zeng, Y., Liu, S., Li, Z., Liu, B., Wu, K., Xiao, J., Yinqi, X., Zheng, Q., Zhang, Y., Yang, H., Wang, J., Smeds, L., Rheindt, F. E., Braun, M., Fjeldsa, J., Orlando, L., Barker, F. K., Jønsson, K. A., Johnson, W., Koepfli, K-P., O’Brien, S., Haussler, D., Ryder, O. A., Rahbek, C., Willerslev, E., Graves, G. R., Glenn, T. C., McCormack, J., Burt, D., Ellegren, H., Alström, P., Edwards, S. V., Stamatakis, A., Mindell, D. P., Cracraft, J., Braun, E. L., Warnow, T., Jun, W., Gilbert, M. T. P. and Zhang, G., 2014. Whole-genome analyses resolve early branches in the tree of life of modern birds. *Science*, **346**, 1320-1331.

Kurochkin, E. N., 1996. A new enantiornithid of the Mongolian Late Cretaceous, and a general appraisal of the infraclass Enantiornithes (Aves). Russian Academy of Sciences: Palaeontological Institute, Moscow, 60pp.

Lapparent, A. F., 1943. Les dinosaures Jurassiques de Damparis (Jura). *Mémoires de la Société Géologique de France, Nouvelle Série*, **47**, 1-21.

Lee, M. S. Y., Cau, A., Naish, D. and Dyke, G. J., 2014a. Morphological clocks in palaeontology, and a mid-Cretaceous origin of crown Aves. *Systematic Biology*, **63**, 442-449.

Lee, M. S. Y., Cau, A., Naish, D. and Dyke, G. J., 2014b. Sustained miniaturization and anatomical innovation in the dinosaurian ancestors of birds. *Science*, **345**, 562-566.

Lloyd, G. T., 2016. Estimating morphological diversity and tempo with discrete character-taxon matrices: implementation, challenges, progress, and future directions. *Biological Journal of the Linnean Society*, **118**, 131-151.

Lloyd, G. T. and Friedman, M., 2013. A survey of palaeontological sampling biases in fishes based on the Phanerozoic record of Great Britain. *Palaeogeography, Palaeoclimatology, Palaeoecology*, **372**, 5-17.

Lloyd, G. T., Davis, K. E., Pisani, D., Tarver, J. E., Ruta, M., Sakamoto, M., Hone, D. W. E., Jennings, R. and Benton, M. J., 2008. Dinosaurs and the Cretaceous Terrestrial Revolution. *Proceedings of the Royal Society of London B*, **275**, 2483-2490.

Meredith, R. W., Janecka, J. E., Gatesy, J., Ryder, O. A., Fisher, C. A., Teeling, E. C., Goodbla, A., Eizirik, E., Simao, T. L. L., Stadler, T., Rabosky, D. L., Honeycutt, R. L., Flynn, J. J., Ingram, C. M., Steiner, C., Williams, T. L., Robinson, T. J., Burk-Herrick, A., Westerman, M., Ayoub, N. A., Springer, M. S., Murphy WJ (2011) Impacts of the Cretaceous Terrestrial Revolution and KPg extinction on mammal diversification. *Science*, **334**, 521-524.

Nesbitt, S. J., 2011. The early evolution of archosaurs: relationships and the origin of major clades. *Bulletin of the American Museum of Natural History*, **352**, 1-292.

Norell, M. A., 1992. Taxic origin and temporal diversity: the effect of phylogeny. Pp. 89–118 in M. J. Novacek and Q. D. Wheeler, eds. *Extinction and Phylogeny*. Columbia University Press, New York.

Paton, T., Haddrath, O. and Baker A. J., 2002. Complete mitochondrial DNA genome sequences show that modern birds are not descended from transitional shorebirds. *Proceedings of the Royal Society of London B*, **269**, 839-846.

Pereira, S. L. and Baker, A. J., 2006. A mitogenomic timescale for birds detects variable phylogenetic rates of molecular evolution and refutes the standard molecular clock. *Molecular Biology and Evolution*, **23**, 1731-1740.

Peters, S. E. and McClennen, M., in press. The Paleobiology Database application programming interface. *Paleobiology*, , .

Pisani, D., Yates, A. M., Langer, M. C. and Benton, M. J., 2002. A genus-level supertree of the Dinosauria. *Proceedings of the Royal Society of London B*, **269**, 915-921.

Prum, R. O., Berv, J. S., Dornburg, A., Field, D. J., Townsend, J. P., Lemmon, E. M. and Lemmon, A. R., 2015. A comprehensive phylogeny of birds (Aves) using targeted next-generation DNA sequencing. *Nature*, **526**, 569-573.

Ragan, M., 1992. Phylogenetic inference based on matrix representation of trees. *Molecular Phylogenetics and Evolution*, **1**, 113-126.

Ruta, M., Wagner, P. J. and Coates, M. I., 2006. Evolutionary patterns in early tetrapods. I. Rapid initial diversification followed by decrease in rates of character change. *Proceedings of Royal Society of London B*, **273**, 2107-2111.

Sakamoto, M. and Ruta, M., 2012. Convergence and divergence in the evolution of cat skulls: temporal and spatial patterns of morphological diversity. *PLoS ONE*, **7**, e39752.

Slack, K. E., Jones, C. M., Ando, T., Harrison, G. L., Fordyce, E., Arnason, U. and Penny, D., 2006. Early penguin fossils, plus mitochondrial genomes, calibrate avian evolution. *Molecular Biology and Evolution*, **23**, 1144-1155.

Smith, A. B., 1994, *Systematics and the Fossil Record: Documenting Evolutionary Patterns*, Blackwell Science, Oxford.

Smith, N. D. and Ksepka, D. T., 2015. Five well-supported fossil calibrations within the "Waterbird" assemblage (Tetrapoda, Aves). *Palaeontologia Electronic*a, **18.1.7FC**, 1-21.

van Tuinen, M., 2009.. Birds (Aves). Pp. 409–411 in *The Timetree of Life*, S. B. Hedges and S. Kumar, Eds. Oxford University Press, Oxford.

Weishampel, D. B., Dodson, P. and Osmólska, H., 2004. *The Dinosauria*, 2nd edn. Berkeley, CA: University of California Press.

Wilkinson, M., 1995. Coping with abundant missing entries in phylogenetic inference using parsimony. *Systematic Biology*, **44**, 501-514.

Wilson, J. A., 2002. Sauropod dinosaur phylogeny: critique and cladistic analysis. *Zoological Journal of the Linnean Society*, **136**, 217-276.

Wright, A. M., Lloyd, G. T. and Hillis, D. M., 2016. Modeling character change heterogeneity in phylogenetic analyses of morphology through the use of priors. *Systematic Biology*, **65**, 602-611.

**7) Supplementary Figure Captions**

**Figure S1** – Schematic workflow for adding new datasets to the metatree. Publications must include a machine-readable version of the character-taxon matrix, or this must either be available elsewhere or recreatable from the information within the publication. Redundancy with older data sets is recorded with “Parent” tags in the XML file, and alternate coding strategies are recorded with the “Sibling” tags.

**Figure S2** – Schematic workflow for generation of the metatree. Greater detail for each step is given in the text.

**Figure S3** – Strict consensus of 1,000 MPTs with STR taxa reinserted. Note the overall high resolution compared to formal supertrees (599 of a maximum possible 961 internal nodes assuming a rooted fully bifurcating tree), with the lowest areas of resolution in Sauropodomorpha and Enantiornithes.

**Figure S4** – Figure to demonstrate that the node-dating algorithm of Hedman is not generalisable to a tree-dating algorithm. Outgroup sequences for three nodes (X-Z) on a hypothetical phylogenetic tree of six fossil taxa (A-F) with sampled ages (T1-T6). Note that for the standard (non-conservative) Hedman approach taxon C is re-dated as T5, the same age as the oldest member (B) of its sister clade (AB), biasing the date of node Y towards a minimum possible age (invalidating the standard approach). Whereas under the conservative Hedman approach taxon C is ignored, as it is younger (T6) than the lower bound for Y set by node AB (T5). Under the conservative scenario the sequence of outgroup taxa for nodes X and Y are identical, meaning branch XY would have zero-length (invalidating the conservative approach). Due to these problems in the present paper only nodes X and Z are considered dateable using the Hedman algorithm (conservative approach), whereas node Y would be dated by making random draws from a uniform distribution bounded by separate random draws from the posterior distributions of nodes X and Z.
